# Increased serum **β**-hydroxybutyrate/acetoacetate ratio and aggravated histological liver inflammation in females with metabolic dysfunction-associated steatotic liver disease and polycystic ovary syndrome

**DOI:** 10.1093/jmcb/mjae048

**Published:** 2024-10-30

**Authors:** Xiaopeng Zhu, Guligeina Aikebaier, Xilei Ban, Qingxia Huang, Hongmei Yan, Xinxia Chang, Xinyu Yang, Xiaoyang Sun, Huiru Tang, Hua Bian, Xin Gao, Mingfeng Xia

**Affiliations:** Department of Endocrinology and Metabolism, Zhongshan Hospital, Fudan Institute for Metabolic Diseases, Fudan University, Shanghai 200032, China; Department of Endocrinology and Metabolism, Zhongshan Hospital, Fudan Institute for Metabolic Diseases, Fudan University, Shanghai 200032, China; Department of Endocrinology and Metabolism, Zhongshan Hospital, Fudan Institute for Metabolic Diseases, Fudan University, Shanghai 200032, China; State Key Laboratory of Genetic Engineering, School of Life Sciences, Human Phenome Institute, Metabonomics and Systems Biology Laboratory at Shanghai International Centre for Molecular Phenomics, Zhongshan Hospital, Fudan University, Shanghai 200438, China; Department of Endocrinology and Metabolism, Zhongshan Hospital, Fudan Institute for Metabolic Diseases, Fudan University, Shanghai 200032, China; Department of Endocrinology and Metabolism, Zhongshan Hospital, Fudan Institute for Metabolic Diseases, Fudan University, Shanghai 200032, China; Department of Endocrinology and Metabolism, Zhongshan Hospital, Fudan Institute for Metabolic Diseases, Fudan University, Shanghai 200032, China; Department of Endocrinology and Metabolism, Zhongshan Hospital, Fudan Institute for Metabolic Diseases, Fudan University, Shanghai 200032, China; State Key Laboratory of Genetic Engineering, School of Life Sciences, Human Phenome Institute, Metabonomics and Systems Biology Laboratory at Shanghai International Centre for Molecular Phenomics, Zhongshan Hospital, Fudan University, Shanghai 200438, China; Department of Endocrinology and Metabolism, Zhongshan Hospital, Fudan Institute for Metabolic Diseases, Fudan University, Shanghai 200032, China; Department of Endocrinology and Metabolism, Zhongshan Hospital, Fudan Institute for Metabolic Diseases, Fudan University, Shanghai 200032, China; Department of Endocrinology and Metabolism, Zhongshan Hospital, Fudan Institute for Metabolic Diseases, Fudan University, Shanghai 200032, China; Department of Endocrinology and Metabolism, Wusong Branch of Zhongshan Hospital, Fudan University, Shanghai 200940, China

Polycystic ovary syndrome (PCOS) is a prevalent endocrine disorder among women of reproductive age, with a global prevalence ranging from 5% to 18% (Shrivastava and Conigliaro, 2023). Currently, a total of 12.13 million cases of infertility (Liu et al., 2024) and 0.24 million cases of cardiovascular disease (Wan et al., 2024) are attributable to PCOS worldwide, and nearly 8 billion US dollars are spent in the USA and Europe for the management of PCOS every year. However, there are still many challenges in understanding and treatment of PCOS.

The precise pathophysiology of PCOS has not been completely elucidated. Recently, a hepato–ovarian axis was suggested through bidirectional two sample Mendelian randomization analysis in large-scale European ancestries, and genetically predicted nonalcoholic fatty liver disease (NAFLD) was identified as a cause of PCOS (Liu et al., 2023). In addition, patients with the metabolic subtype of PCOS exhibit a reduction in liver-derived sex hormone-binding globulin, which is downregulated in the presence of NAFLD and functionally leads to enhanced bioavailability of testosterone to the ovaries (Urbano et al., 2022). Metabolic dysfunction-associated steatotic liver disease (MASLD) is a new nomenclature for NAFLD that has been described as the hepatic manifestation of metabolic syndrome and a continuum from obesity to a series of metabolic disorders, including PCOS. Due to the close connection between MASLD and PCOS, a large percentage of women of reproductive age are clinically diagnosed with both PCOS and MASLD (Vassilatou et al., 2015). However, the liver histological and metabolic features in female MASLD patients with PCOS, compared with those in female MASLD patients without PCOS, are not fully understood.

To explore this issue, we included 225 women with biopsy-proven MASLD from the Department of Endocrinology and Metabolism, Zhongshan Hospital, Fudan University, China, in this study (Supplementary Figure  S1). Among these patients, 28 (12.4%) MASLD patients were diagnosed with PCOS according to the Rotterdam criteria (Figure 1A). Liver tissue sections from MASLD patients with or without PCOS were examined by haematoxylin and eosin and reticulin staining (Figure 1B). Compared with female MASLD patients without PCOS, female patients with both MASLD and PCOS presented with more severe liver steatosis (*P* = 0.031) and lobular inflammation (*P* = 0.028) (Figure 1C and D). However, other liver histological features, such as ballooning degeneration and fibrosis grade, were not significantly different between the two groups (Figure 1E and F). Notably, patients with PCOS were much younger and had higher body mass indexes (BMIs) than those without PCOS (*P *< 0.001) (Supplementary Table S1), which might confound the correlation between the presence of PCOS and the grade of liver steatosis and inflammation. Thus, age, BMI, cigarette smoking status, and alcohol consumption status were further adjusted in the statistical model (see Supplementary material for details). After these adjustments, MASLD patients with PCOS still had a higher risk of aggravated liver lobular inflammation (odds ratio [OR], 2.77; 95% confidence interval [CI], 1.06–7.23) but not an increased risk of liver steatosis compared to those without PCOS (Supplementary Figure S2A).

**Figure 1:**
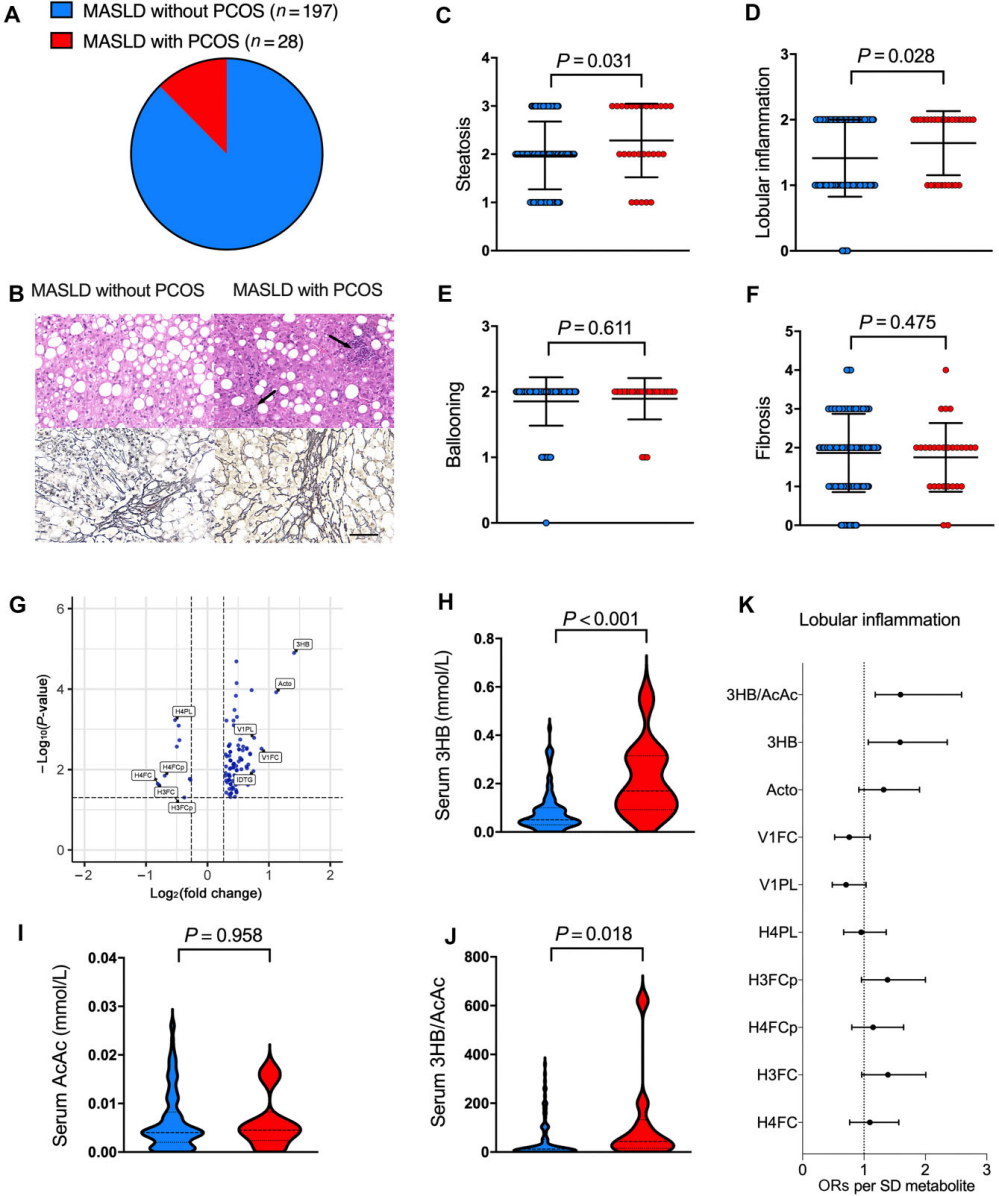
Liver histological and serum metabolomic features of MASLD patients with PCOS. (**A**) The percentage of patients with PCOS in the study cohort. (**B**) Representative H&E and reticular staining of liver sections. Scale bar, 100 μm. (**C**–**F**) Comparison of the grades of histological liver steatosis (**C**), lobular inflammation (**D**), ballooning (**E**), and fibrosis (**F**) between female MASLD patients with and without PCOS. (**G**) Volcano plot of metabolites differentially expressed in patients with both MASLD and PCOS. (**H**–**J**) Comparison of serum3HB (**H**) and AcAc (**I**) levels and the 3HB/AcAc ratio (**J**) between female MASLD patients with and without PCOS. (**K**) The ORs per standard deviation (SD) metabolite for greater risk of aggravated liver lobular inflammation. All tests were two-sided, and *P* < 0.05 was considered statistically significant.

Previous studies have indicated that insulin resistance precedes MASLD progression to metabolic dysfunction-associated steatohepatitis (MASH) and liver fibrosis (Anstee et al., 2013). Despite the similar serum glucose and lipid metabolic parameters in the study, fasting C-peptide, fasting insulin, homeostatic model assessment for insulin resistance (HOMA-IR), and androgen levels were notably higher in patients with both MASLD and PCOS than in MASLD patients without PCOS (*P *< 0.001) (Supplementary Table S1). To exclude the potential confounding effect of insulin resistance and other metabolic parameters, a 1:1 propensity score matching strategy was used to identify 28 pairs of MASLD patients (with vs. without PCOS) who were matched strictly for age, BMI, and HOMA-IR level (Supplementary Figure S1). There was no difference in the demographic or metabolic parameters or sex hormone levels between the two groups after propensity score matching. Compared with MASLD patients without PCOS, patients with both MASLD and PCOS had significantly higher liver lobular inflammation (*P* = 0.015), histological activity (*P* = 0.030), and steatosis, activity, and fibrosis (SAF) score (*P* = 0.028) (Supplementary Figure S3). Even after multivariate adjustment, patients with both MASLD and PCOS still showed greater liver lobular inflammation (OR, 3.69; 95% CI, 1.15–11.80), histological activity (OR, 3.09; 95% CI, 1.01–9.41), and SAF (OR, 3.30; 95% CI, 1.19–9.12) compared to matched patients without PCOS (Supplementary Figure S2B). Therefore, the severe liver inflammation in patients with both MASLD and PCOS could not be completely attributed to their adiposity and insulin resistance status.

Metabolomics offers a powerful tool for revealing the complex metabolic changes underlying diseases, ultimately paving the way for further exploration of disease associations involving the hepato–ovarian axis. Inspired by our previous findings of outstanding changes in the metabolomic profiles of sarcopenia patients (Zhu et al., 2023) and patients with MASLD associated with two genes (*PNPLA3* and *TM6SF2*) (Xia et al., 2021; Zhu et al., 2023), we analysed serum metabolomic profiles of female MASLD patients with or without PCOS. Up to 92 serum metabolites were upregulated and 11 serum metabolites were downregulated in the PCOS group (Supplementary Table S2). The top 5 upregulated and downregulated metabolites were labelled in a volcano plot (Figure 1G), among which β-hydroxybutyrate (3HB) was most significantly upregulated, with >2-fold higher concentration in patients with both MASLD and PCOS than in MASLD patients without PCOS (0.22 ± 0.15 vs. 0.08 ± 0.07 mmol/L, *P* < 0.001) (Figure 1H). However, serum levels of another ketone body, acetoacetate (AcAc), were not significantly different between the two groups (0.008 ± 0.006 vs. 0.008 ± 0.005 mmol/L, *P* = 0.958) (Figure 1I), thus leading to the increased serum 3HB/AcAc ratio, a biomarker of the hepatic mitochondrial redox state, in patients with both MASLD and PCOS (39.2 ± 34.5 vs. 24.6 ± 36.4, *P* = 0.018) (Figure 1J). The difference in serum 3HB/AcAc ratio remained significant, even after adjustment for liver SAF scores (*P* = 0.025). In addition, among the 355 metabolites measured using a 600 MHz AVANCE III NMR spectrometer equipped with a BBI probe (Bruker Biospin GmbH) (Supplementary Table S3), patients with both MASLD and PCOS exhibited significantly higher serum levels of phospholipids and free cholesterol in VLDL-1 (V1PL and V1FC) but significantly lower serum levels of free cholesterol in HDL-3 (H3FC), free cholesterol in HDL-4 (H4FC), and phospholipids in HDL-4 (H4PL), compared to MASLD patients without PCOS (Supplementary Table S2).

We further investigated whether the altered serum metabolites in patients with both MASLD and PCOS were associated with the severer histological liver inflammation. Among the top upregulated and downregulated metabolites, only serum 3HB and the 3HB/AcAc ratio were significantly associated with higher grades of liver lobular inflammation (Figure 1K). Further quantitative correlation analysis revealed that serum 3HB/AcAc ratio was associated with liver lobular inflammation (*r* = 0.220, *P* = 0.016) and ballooning (*r* = 0.199, *P* = 0.029) but not with steatosis (*r* = 0.059, *P* = 0.522) or fibrosis (*r* = 0.084, *P* = 0.362) (Supplementary Figure S4). A recent study indicated that genetic risk factors contribute to the development and progression of MASLD mainly through the impairment of hepatic mitochondrial function, evidenced by an increase in the 3HB/AcAc ratio (Luukkonen et al., 2022). Intriguingly, in the present study, patients with both MASLD and PCOS were characterized by an increase in the 3HB/AcAc ratio and an increase in the risk of histological liver inflammation and activity, coincident with that MASLD was caused by genetic risk factors. These findings underscore the relationship between the hepatic mitochondrial redox state and histological liver inflammation observed in MASLD patients with PCOS, highlighting potential implications for adverse liver outcomes in these patients. Further studies are still required to validate the results in larger female cohorts with liver biopsy.

Overall, this study sheds light on the liver histological characteristics of women with both MASLD and PCOS, revealing the severer liver inflammation and distinct metabolomic profiles. These profiles are marked by a dysregulated mitochondrial redox status, as indicated by the increased 3HB/AcAc ratio. Therefore, our study indicates the necessity of screening for MASH in premenopausal women with both MASLD and PCOS and treating these patients with a tailored management strategy, given the unique mechanism involving dysregulated mitochondrial redox status.

*[This study was supported by the National Key Research and Development Program of China (2021YFC2700403), Shanghai Municipal Science and Technology Major Project (2023SHZDZX02), the National Natural Science Foundation of China (81873660 and 32371333), and the Science and Technology Commission of Shanghai Municipality (23XD1423300 and 23ZR1411000).]*

## Supplementary Material

mjae048_Supplemental_File
