# Supplementary material for: Increased serum β-hydroxybutyrate/acetoacetate ratio and aggravated histological liver inflammation in females with metabolic dysfunction-associated steatotic liver disease and polycystic ovary syndrome
Source: J Mol Cell Biol. 2024 Oct 30;16(11):mjae048. doi: 10.1093/jmcb/mjae048 (PMC12096068; doi:10.1093/jmcb/mjae048)
Supplement: mjae048_Supplemental_File [file mjae048_supplemental_file.pdf]

Supplementary material

Increased serum β-hydroxybutyrate/acetoacetate ratio and aggravated histological liver inflammation in females with metabolic dysfunction-associated steatotic liver disease and polycystic ovary syndrome

Xiaopeng Zhu[1,†], Guligeina Aikebaier[1,†], Xilei Ban[1,†], Qingxia Huang[2], Hongmei Yan[1], Xinxia Chang[1], Xinyu Yang[1], Xiaoyang Sun[1], Huiru Tang[2], Hua Bian[1,*], Xin Gao[1,*], and Mingfeng Xia[1,3,*]

[1]Department of Endocrinology and Metabolism, Zhongshan Hospital, Fudan Institute for Metabolic Diseases, Fudan University, Shanghai 200032, China

[2]State Key Laboratory of Genetic Engineering, School of Life Sciences, Human Phenome Institute, Metabonomics and Systems Biology Laboratory at Shanghai International Centre for Molecular Phenomics, Zhongshan Hospital, Fudan University, Shanghai 200438, China

[3]Department of Endocrinology and Metabolism, Wusong Branch of Zhongshan Hospital, Fudan University, Shanghai 200940, China

[†]These authors contributed equally to this work.

*Corresponding authors

Supplementary Materials and methods

Patients

The participants were from the fatty liver clinic of Zhongshan Hospital, Fudan University. Patients diagnosed with fatty liver by ultrasonography who had indications of liver biopsy, such as diabetes, obesity, or an elevated level of liver enzymes, were suggested to undergo liver biopsy according to the EASL–EASD–EASO clinical practice guidelines (European Association for the Study of the et al., 2016). The inclusion criterion was biopsy-proven fatty liver. The exclusion criteria were as follows: (i) type 1 diabetes mellitus, gestational diabetes, and other specific types of diabetes; (ii) acute complications of diabetes; (iii) liver disease caused by other factors such as chronic viral hepatitis; (iv) hypothyroidism; (v) excessive use of alcohol (more than 20 g/day for men, more than 10 g/day for women); (vi) use of drugs that affect glucose metabolism; (vii) severe renal disease or abnormal renal function (Cr ≥115 μmol/L); and (viii) history of malignant tumor, severe mental illness, or parenteral nutrition. The competing etiologies of steatosis were ruled out based on laboratory examination and liver histology.

The diagnosis of PCOS is made according to the Rotterdam criteria (Rotterdam, 2004). A total of 225 female subjects with liver biopsy-proved MASLD, including 28 PCOS subjects and 197 non-PCOS, were enrolled in this study. A 1:1 propensity score matching was conducted to select 28 PCOS subjects and 28 non-PCOS strictly matched with age, BMI and insulin resistance levels for subgroup analysis All protocols were performed in accordance with the Declaration of Helsinki of 1975 and approved by the Ethics Committee of the Zhongshan Hospital, Fudan University, and each subject provided written informed consent.

Evaluation of liver histology

Liver biopsy was performed to determine the severity of liver steatosis, inflammation, and fibrosis. SAF scores were applied to assess the severity of MASLD (Bedossa and Consortium, 2014; Kleiner et al., 2005).

Anthropometric and biochemical measurements

All subjects underwent routine anthropometric measurements, serum biochemical examinations, and medical history analyses. Standing height and body weight were measured without shoes and outer clothing, and the body mass index (BMI) was calculated as weight (kg) divided by standing height squared (m^2). Blood samples were collected after a fasting period of at least 12 hours. Serum total cholesterol (TC), triglycerides (TGs), high-density lipoprotein cholesterol (HDL-c), low-density lipoprotein cholesterol (LDL-c), and liver enzymes (alanine aminotransferase [ALT], aspartate aminotransferase [AST], and gamma-glutamyl transferase [GGT]) levels were measured using a model 7600 automated bioanalyzer (Hitachi, Tokyo, Japan). Fasting blood glucose, the 2-hour OGTT glucose following a 75-g glucose OGTT or a standard meal were measured using the glucose oxidase method. Fasting C-peptide and fasting insulin were measured via electrochemiluminescence. HbA1c was assessed via high-performance liquid chromatography.

Serum metabolomic measurements by ^{1}H nuclear magnetic resonance spectroscopy (^{1}H‐NMR)

A 600 MHz NMR spectrometer with a BBI probe (Bruker Biospin, Germany) was used to conduct 1H-NMR analysis, as reported previously (Jimenez et al., 2018; Xia et al., 2022). Metabolites with those exhibiting a missing rate greater than 20% were excluded. Any remaining missing value was replaced with half of the minimum value. Subsequently, a total of 355 quantitative metabolites (157 directly measured and 198 calculated) were considered for analysis, including lipoprotein parameters, fatty acid ratios, and small metabolites such as amino acids, ketone bodies, glucose, carboxylic acids, and acute-phase N-acetyl-glycoproteins (NAG), as detailed in Table S3. Lipoprotein parameters were measured in mg/dL, while other metabolomic parameters were measured in mmol/L.

Statistical analysis

All statistical analyses were performed using SPSS software version 26.0 (SPSS, Chicago, IL). All data were tested for normality and homogeneity of variance. Normally distributed data are shown as the mean ± SD, whereas non-normally distributed data are shown as the median followed by the 25th and 75th percentiles. The independent two-sample t-test or the Mann-Whitney U test was used for comparisons of continuous data, and the chi-squared test was used for comparisons of categorical variables. Ordinal or logistic regression analysis was applied to evaluate the odds ratios (ORs) of the patients with both MASLD and PCOS for liver histological steatosis, lobular inflammation, ballooning, fibrosis grades and a series of metabolic diseases, after adjustment for age, BMI, cigarette smoking and alcohol drinking. Differential expressed metabolites were analyzed using MetaboAnalyst (www.metaboanalyst.ca/MetaboAnalyst/) online. Metabolites with fold change >1.2 and p<0.05 were selected for subsequent analysis. We also analyzed the associations of the selected ^{1}H-NMR-based metabolites with the grades of liver histological features using ordinal regression models. The correlations between serum 3HB/AcAc ratios and liver histological steatosis, lobular inflammation, ballooning, activity and fibrosis grades were analyzed using Spearman correlation analysis. All tests were two-sided, and p<0.05 was considered statistically significant unless otherwise stated.

Supplementary References

Bedossa, P., and Consortium, F.P. (2014). Utility and appropriateness of the fatty liver inhibition of progression (FLIP) algorithm and steatosis, activity, and fibrosis (SAF) score in the evaluation of biopsies of nonalcoholic fatty liver disease. Hepatology *60*, 565-575.

European Association for the Study of the, L., European Association for the Study of, D., and European Association for the Study of, O. (2016). EASL-EASD-EASO Clinical Practice Guidelines for the management of non-alcoholic fatty liver disease. J Hepatol *64*, 1388-1402.

Jimenez, B., Holmes, E., Heude, C., Tolson, R.F., Harvey, N., Lodge, S.L., Chetwynd, A.J., Cannet, C., Fang, F., Pearce, J.T.M.*, et al.* (2018). Quantitative Lipoprotein Subclass and Low Molecular Weight Metabolite Analysis in Human Serum and Plasma by (1)H NMR Spectroscopy in a Multilaboratory Trial. Anal Chem *90*, 11962-11971.

Kleiner, D.E., Brunt, E.M., Van Natta, M., Behling, C., Contos, M.J., Cummings, O.W., Ferrell, L.D., Liu, Y.C., Torbenson, M.S., Unalp-Arida, A.*, et al.* (2005). Design and validation of a histological scoring system for nonalcoholic fatty liver disease. Hepatology *41*, 1313-1321.

Rotterdam, E.A.-S.P.c.w.g. (2004). Revised 2003 consensus on diagnostic criteria and long-term health risks related to polycystic ovary syndrome (PCOS). Hum Reprod *19*, 41-47.

Xia, M., Ma, S., Huang, Q., Zeng, H., Ge, J., Xu, W., Wu, Q., Wu, L., Li, X., Ma, H.*, et al.* (2022). NAFLD-related gene polymorphisms and all-cause and cause-specific mortality in an Asian population: the Shanghai Changfeng Study. Aliment Pharmacol Ther *55*, 705-721.

Supplementary Figures

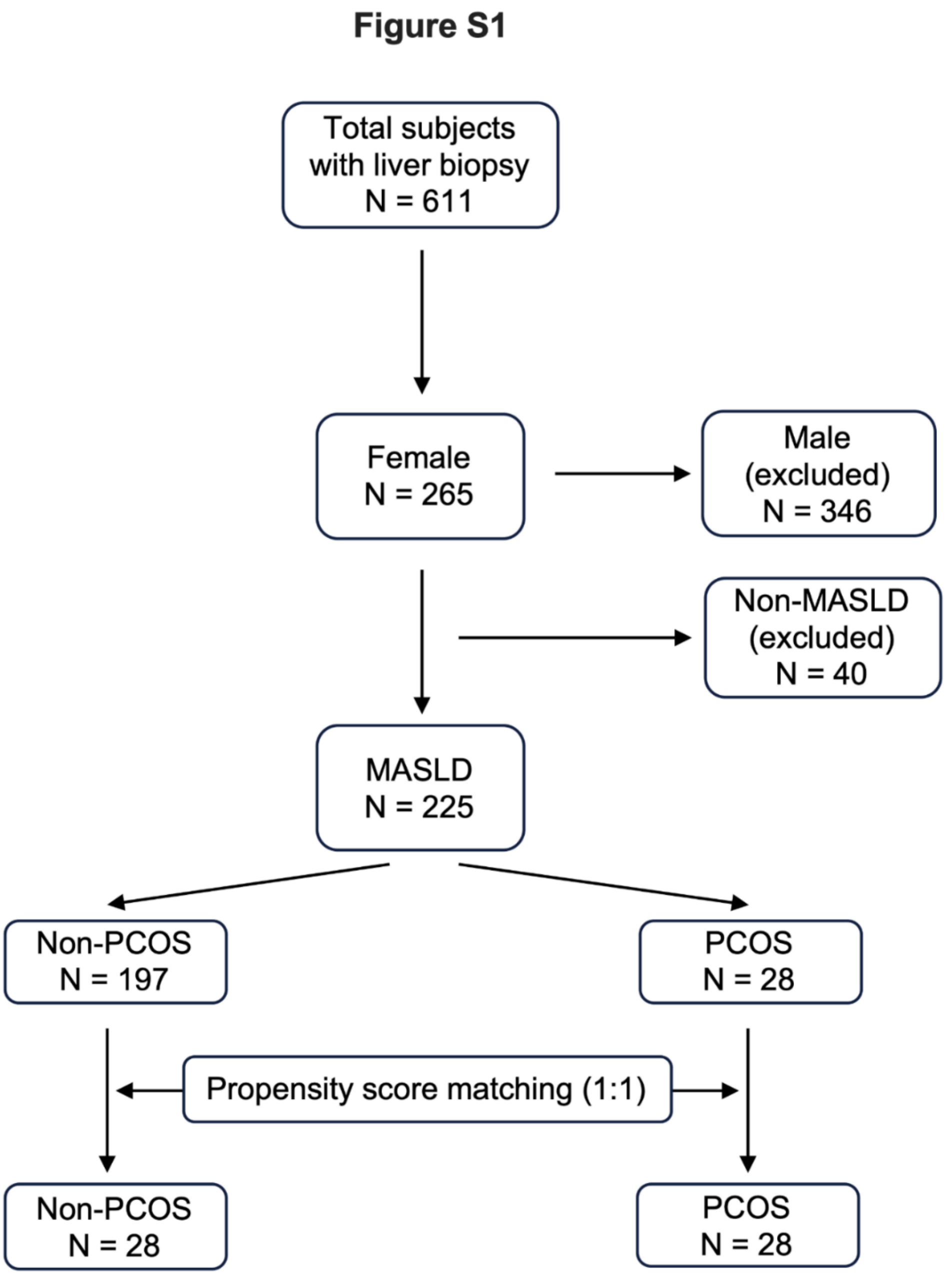

Supplementary Figure S1 The participant flow diagram.

Figure S2

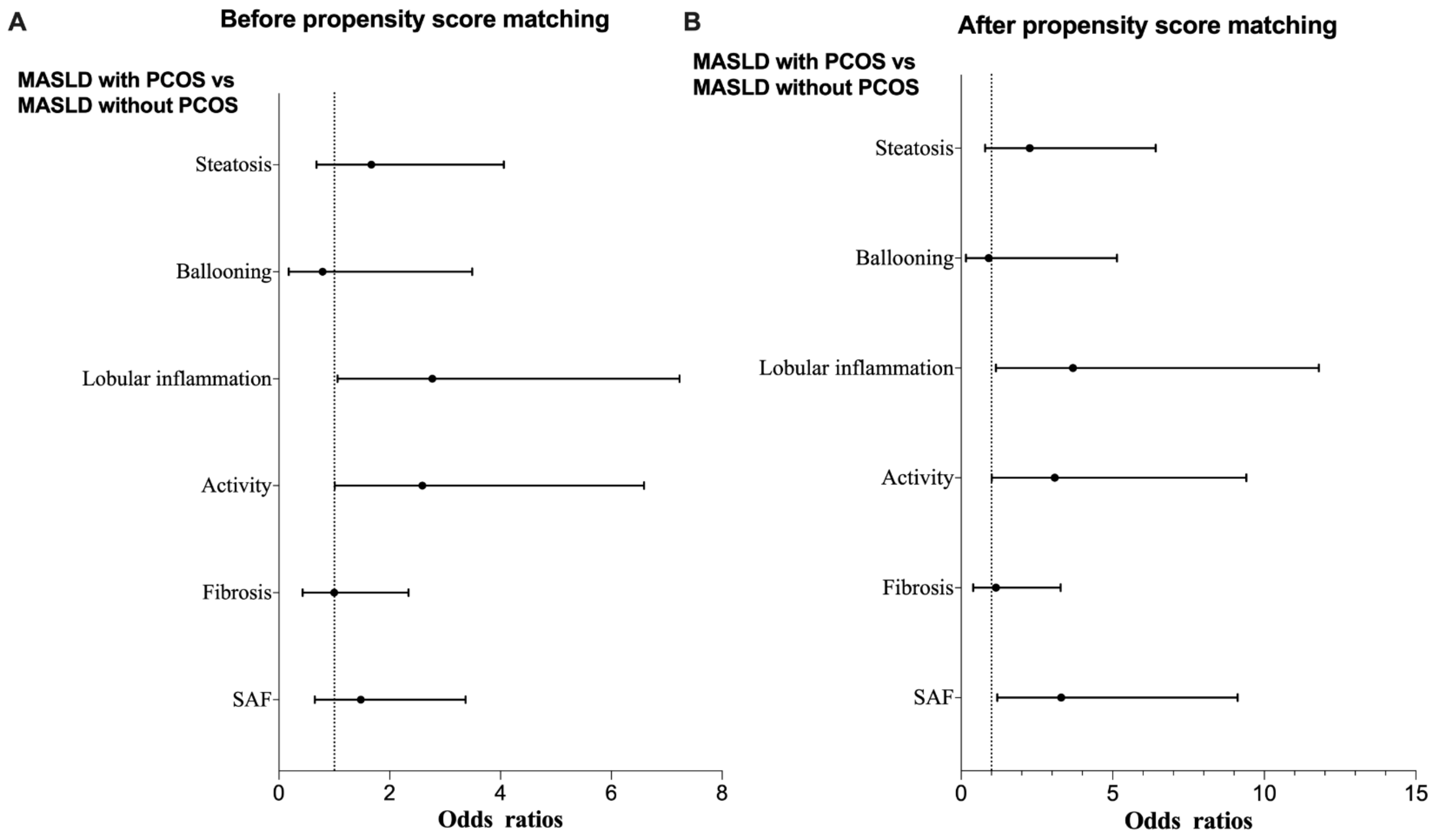

Supplementary Figure S2 Associations of the presence of PCOS with liver histological features among females with MASLD. The odds ratios (ORs) for liver steatosis, ballooning, lobular inflammation, activity, fibrosis, and SAF scores related to the presence of PCOS in MASLD patients before (**A**) and after (**B**) propensity score matching.

Figure S3

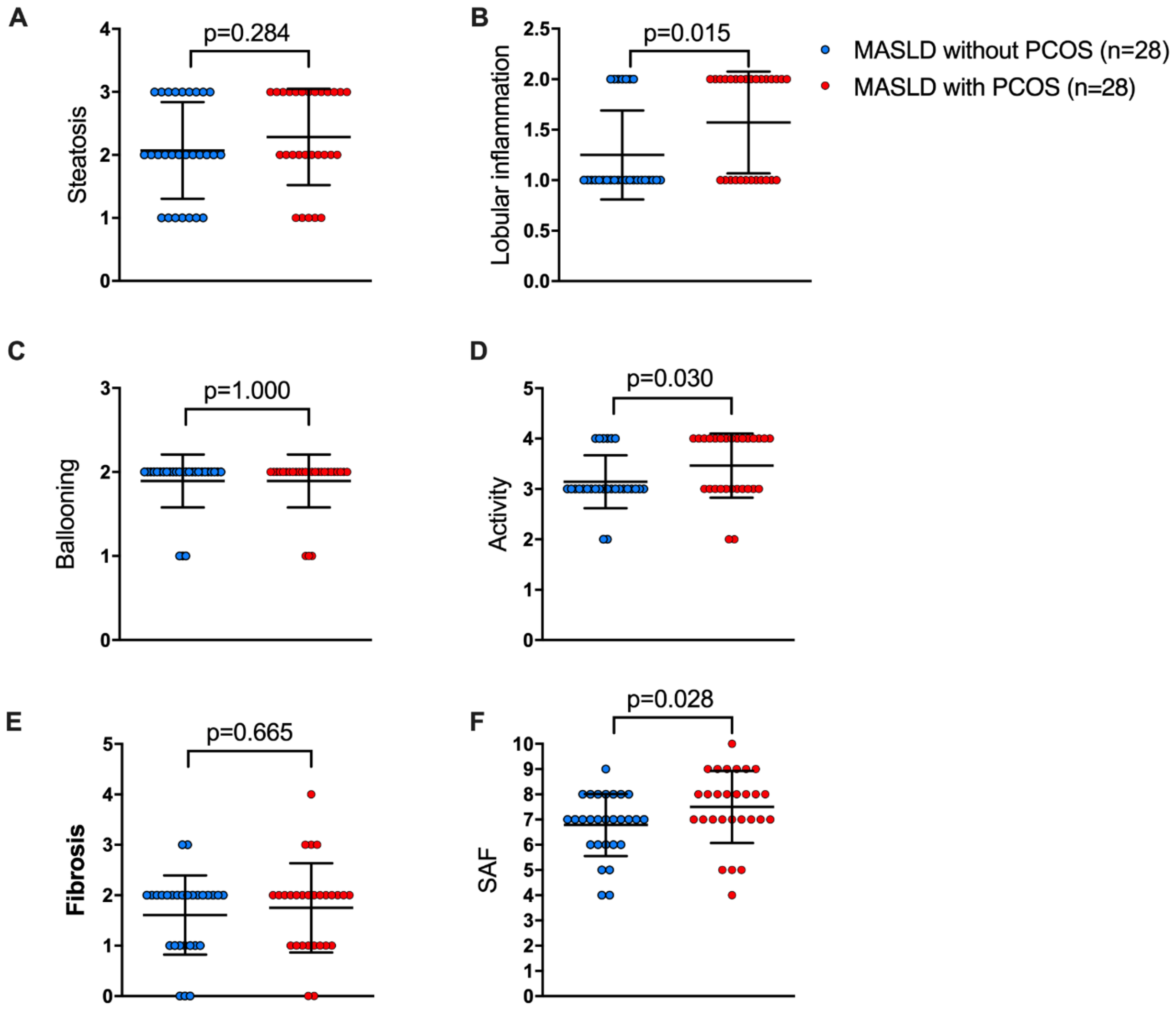

Supplementary Figure S3 The liver histology in female MASLD patients with or without PCOS after propensity score matching. Comparison of liver histological steatosis (**A**), lobular inflammation (**B**), ballooning (**C**), activity (**D**), fibrosis (**E**), and SAF (**F**) between the female MASLD patients with or without PCOS strictly matched with age, BMI, and HOMA-IR levels.

Figure S4

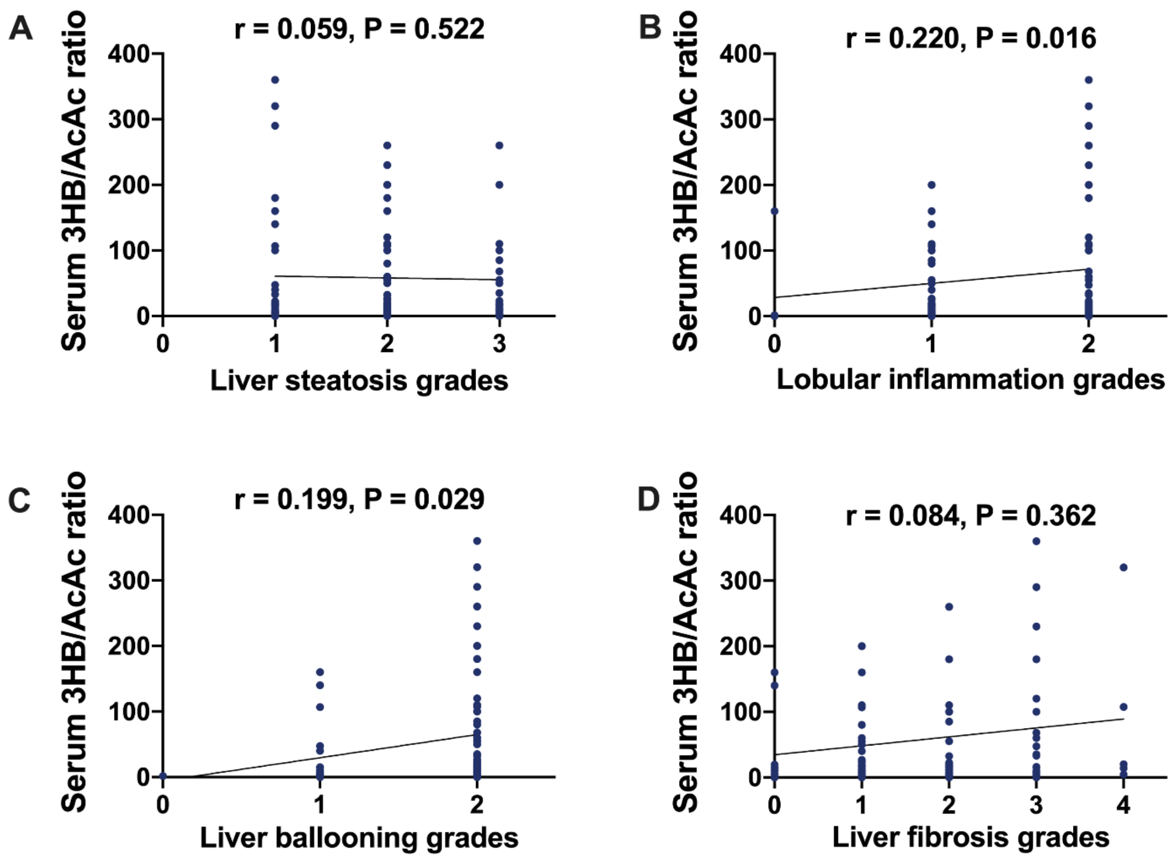

Supplementary Figure S4 Correlation of serum β-hydroxybutyrate/acetoacetate (3HB/AcAc) ratio with liver histological features, i.e. liver steatosis (**A**), lobular inflammation (**B**), ballooning (**C**), and fibrosis (**D**) grades.

Supplementary Tables

Supplementary Table S1 Clinical characteristics of the MASLD patients with and without PCOS.

	Before propensity score matching				After propensity score matching			
	**Total (N=225)**	**Non-PCOS (N=197)**	**PCOS (N=28)**	***P*-value**	**Total (N=56)**	**Non-PCOS (N=28)**	**PCOS (N=28)**	***P*-value**
Age, years	48±15	51±14	29±9	<0.001	48±15	29±5	29±9	0.820
BMI, kg/m^2	29.2±5.6	28.3±5.0	35.2±5.9	<0.001	29.2±5.6	33.3±5.9	35.2±5.9	0.286
Alcohol drinking, n (%)	2 (0.9%)	1(0.5%)	1(3.6%)	0.106	1 (1.8%)	0 (0.0%)	1 (3.6%)	0.313
Cigarette smoking, n (%)	1 (0.4%)	0 (0.0%)	1 (3.6%)	0.008	1 (1.8%)	0 (0.0%)	1 (3.6%)	0.313
FPG, mmol/L	5.8±1.8	5.9±1.8	5.3±1.5	0.101	5.8±1.8	5.7±1.6	5.3±1.5	0.385
2hPG, mmol/L	12.3±4.1	12.5±4.1	11.1±3.6	0.124	12.3±4.1	10.8±3.4	11.1±3.6	0.840
HbA1c, %	6.9±1.6	7.0±1.7	6.2±1.1	0.054	6.9±1.6	6.6±2.0	6.2±1.1	0.461
FCP, ng/ml	3.1 (2.6-3.9)	3.0 (2.5-3.7)	4.2 (3.2-5.2)	<0.001	4.0 (3.1-4.8)	3.6 (3.0-4.4)	4.2 (3.2-5.2)	0.245
Fins, mU/L	15.0 (11.0-20.5)	14.4 (10.6-18.7)	24.4(17.8-36.4)	<0.001	20.1 (15.0-29.2)	15.7 (12.0-23.5)	24.4(17.8-36.4)	0.073
TC, mmol/L	4.8±1.1	4.7±1.1	5.0±1.0	0.254	4.8±1.1	4.6±1.0	5.0±1.0	0.280
TG, mmol/L	1.72 (1.23-2.27)	1.72 (1.23-2.28)	1.65 (1.33-2.27)	0.789	1.62 (1.16-2.42)	1.60 (1.10-2.58)	1.65 (1.33-2.27)	0.505
HDL-c, mmol/L	1.13±0.28	1.15±0.28	1.05±0.23	0.096	1.13±0.28	1.14±0.28	1.05±0.23	0.235
LDL-c, mmol/L	2.76±0.95	2.71±0.93	3.07±1.07	0.073	2.76±0.95	2.67±0.96	3.07±1.07	0.210
ALT, U/L	64 (36-96)	61 (35-93)	79 (38-113)	0.127	67 (36-104)	62 (31-84)	79 (38-113)	0.203
AST, U/L	41 (27-65)	41 (27-63)	44 (27-75)	0.243	40 (26-65)	39 (21-52)	44 (27-75)	0.346
GGT, U/L	48 (31-75)	47 (30-72)	68 (34-92)	0.082	56 (30-79)	48 (25-64)	68 (34-92)	0.097
HOMA-IR	3.82 (2.63-5.52)	3.58 (2.54-5.12)	5.45 (3.56-7.75)	0.001	5.32 (3.23-6.78)	3.91 (2.98-6.29)	5.45 (3.56-7.75)	0.113
Androgen, nmol/L	0.96±0.81	0.82±0.77	1.58±0.66	<0.001	1.48±0.64	1.36±0.60	1.58±0.66	0.290
Estrogen, pmol/L	149.6±249.2	138.5±265.7	165.6±84.3	0.653	257.6±323.6	365.8±451.8	165.6±84.3	0.090
Progesterone, nmol/L	1.65±4.20	1.48±4.00	2.44±5.07	0.354	2.55±4.99	2.68±5.04	2.44±5.07	0.887

BMI = BMI body mass index; FPG = fasting plasma glucose; 2hPG = 2 hours postprandial plasma glucose; HbA1c = hemoglobin A1c; FCP = fasting plasma C-peptide; FIns = fasting plasma insulin; TC = total cholesterol; TG = triglyceride; HDL-c = high density lipoprotein cholesterol; LDL-c = low density lipoprotein cholesterol; ALT = alanine aminotransferase; AST = aspartate aminotransferase; GGT = gamma-glutamyl transferase; HOMA-IR = homeostatic model assessment for insulin resistance.

Supplementary Table S2 Metabolits differentially expressed in patients with MASLD and PCOS.

Metabolites	t.stat	*P*-value	FDR	Fold change	Log_2(fold change)
3HB	-4.5595	1.26E-05	0.001924	2.6574	1.41
Acto	-3.9799	0.00012	0.006075	2.1719	1.1189
3HB/AcAc	-2.3954	0.017618	0.063582	2.1199	1.084
Succ	-2.565	0.029	0.091185	2.039	1.02786
V1FC	-3.0272	0.003032	0.027547	1.8403	0.87991
V1PL	-3.2251	0.00163	0.023668	1.6931	0.75967
IDTG	-2.5853	0.010946	0.049095	1.6792	0.74776
ICHHCR	-4.0117	0.000106	0.006075	1.6464	0.71928
V1TG	-2.9216	0.004174	0.031288	1.6186	0.69471
V1LP	-2.9356	0.004003	0.031288	1.6168	0.69311
V2FC	-2.7212	0.007489	0.041649	1.586	0.66536
V2TG	-2.2855	0.024068	0.075677	1.578	0.65814
V1CH	-2.6464	0.009244	0.044752	1.5625	0.64382
IDLP	-3.039	0.002924	0.027547	1.558	0.63972
IDPL	-3.0224	0.003076	0.027547	1.5563	0.63812
V2PL	-2.6805	0.008404	0.044194	1.5533	0.63537
V2LP	-2.4729	0.014826	0.058726	1.5454	0.62794
H4TGp	-3.3707	0.001014	0.01718	1.5281	0.61176
IDCE	-3.0674	0.002679	0.027374	1.4962	0.58134
I0CH	-2.997	0.003324	0.028162	1.4894	0.57476
V3FC	-2.4347	0.016399	0.064125	1.4841	0.56962
V1CE	-2.4202	0.017036	0.064367	1.4807	0.56624
TGHCR	-3.3094	0.00124	0.018914	1.4697	0.55553
I0FC	-2.8165	0.005692	0.037277	1.4695	0.55528
VIDTG	-2.8212	0.005615	0.037277	1.4675	0.55336
V2CH	-2.6247	0.009821	0.046084	1.466	0.55192
VILTG	-3.1949	0.001795	0.024676	1.4476	0.53368
V0TG	-2.8496	0.005167	0.035818	1.4461	0.5322
V3CH	-2.3157	0.022303	0.073949	1.4339	0.51991
IDAB	-3.0135	0.003161	0.027547	1.4279	0.51385
IDPN	-3.0138	0.003158	0.027547	1.4278	0.51383
VCHHCR	-2.7837	0.00626	0.037439	1.4224	0.50836
TGall	-3.0893	0.002502	0.027374	1.4149	0.50073
V0LP	-2.6081	0.010281	0.047512	1.3921	0.47722
VIDCH	-2.6019	0.010459	0.047613	1.3901	0.4752
nHCHCR	-4.1187	7.10E-05	0.005414	1.3898	0.47483
V3CE	-2.1067	0.037261	0.10203	1.3891	0.47411
V3PL	-2.1294	0.035296	0.097867	1.3867	0.4717
ABHCR	-4.4391	2.05E-05	0.002079	1.3862	0.4711
TG	-2.6917	0.008142	0.044194	1.3836	0.46843
ABA1R	-3.9249	0.000146	0.006376	1.3783	0.46285
L1TG	-2.1641	0.032473	0.092565	1.3782	0.46277
V0CE	-2.2873	0.023957	0.075677	1.3768	0.46133
L0TG	-3.4141	0.000878	0.015751	1.3744	0.45884
V2CE	-2.002	0.047577	0.124	1.3637	0.4475
VLCHHCR	-3.7005	0.000328	0.011436	1.3567	0.44008
L5TG	-2.4996	0.013808	0.057952	1.3535	0.43672
VIDAB	-2.738	0.007139	0.041156	1.3467	0.4294

VIDPN	-2.7374	0.007152	0.041156	1.3466	0.42933
L6TG	-3.4452	0.000791	0.015433	1.345	0.42764
nH0CHp	-3.5243	0.000605	0.014196	1.3414	0.42378
L2PN	-2.6582	0.008947	0.044752	1.3387	0.42082
L2AB	-2.6576	0.00896	0.044752	1.3386	0.42073
LHCR	-2.7202	0.007511	0.041649	1.3372	0.41919
V0CH	-2.217	0.028541	0.08705	1.3349	0.41672
VIDFC	-2.374	0.019212	0.066538	1.3295	0.41089
VIDPL	-2.2292	0.027694	0.085321	1.3226	0.40337
FA	-3.103	0.002	0.022211	1.3221	0.40283
L2TG	-2.1817	0.031115	0.090381	1.3186	0.39897
L5AB	-2.087	0.039039	0.10456	1.306	0.38515
L5PN	-2.0865	0.039083	0.10456	1.3059	0.38508
V0AB	-2.304	0.022973	0.073949	1.2986	0.37691
TGPLR	-2.6477	0.009212	0.044752	1.2985	0.37687
V0PN	-2.3029	0.023033	0.073949	1.2985	0.3768
L6AB	-2.7991	0.005989	0.037277	1.2984	0.37678
L6PN	-2.7992	0.005986	0.037277	1.2984	0.37678
L2CH	-1.9966	0.048169	0.124	1.2971	0.37525
VILAB	-2.9684	0.003626	0.029117	1.2932	0.37097
VILPN	-2.9682	0.003628	0.029117	1.2932	0.37095
L2FC	-2.8065	0.00586	0.037277	1.2921	0.36969
NAG1	-2.6349	0.009546	0.045492	1.2868	0.36382
H3TGp	-3.1095	0.00235	0.027374	1.2862	0.36308
V3CHTGR	-2.4777	0.014638	0.058726	1.2857	0.36252
L2LP	-2.1939	0.030199	0.088913	1.2811	0.35742
VILCH	-2.8945	0.004526	0.032864	1.2803	0.35651
V0FC	-2.0557	0.042021	0.11145	1.2799	0.35605
L0AB	-2.3949	0.018196	0.065298	1.2795	0.35556
L0PN	-2.3949	0.018198	0.065298	1.2795	0.35555
L6CE	-2.5142	0.013277	0.057766	1.2762	0.35187
L6CH	-2.3995	0.017981	0.065298	1.272	0.34706
ABPN	-3.0777	0.002595	0.027374	1.2719	0.34694
ApoB	-3.0776	0.002595	0.027374	1.2719	0.34693
L6LP	-2.4937	0.014028	0.057952	1.2713	0.34631
L6PL	-2.3443	0.020737	0.070276	1.2481	0.31973
VILFC	-2.8826	0.004687	0.033248	1.2446	0.31572
CHHCR	-3.5243	0.000605	0.014196	1.2386	0.30876
L0LP	-2.1044	0.037465	0.10203	1.2362	0.30593
nH0CH	-2.3817	0.018832	0.06602	1.2341	0.30342
VILPL	-2.6822	0.008363	0.044194	1.2329	0.3021
V4FCp	-2.5445	0.012233	0.054074	1.2277	0.29591
Ile	-2.9191	0.004206	0.031288	1.2271	0.29524
NAG2	-2.4878	0.014251	0.057952	1.2213	0.2884
H3CH	2.3992	0.017997	0.065298	0.82568	-0.27634
H4A2	2.4189	0.017094	0.064367	0.81912	-0.28786
H3CHTGR	1.9848	0.04949	0.12475	0.77053	-0.37608
H4CE	3.1835	0.001861	0.024676	0.72857	-0.45687
H4LP	3.4383	0.00081	0.015433	0.7226	-0.46874
H4CH	3.0657	0.002693	0.027374	0.70775	-0.49868
H4PL	3.533	0.000588	0.014196	0.69387	-0.52726

H3FCp	2.0504	0.042543	0.11186	0.67617	-0.56454
H4FCp	2.4887	0.014215	0.057952	0.61744	-0.69563
H3FC	2.2752	0.024701	0.076876	0.58034	-0.78504
H4FC	2.3108	0.022578	0.073949	0.57103	-0.80836

Supplementary Table S3 List of metabolites measured in Chinese cohort (N=355).

Classification	Abbreviation	Full name	Unit	Direct measurement	Class1
Auto Report	TG	Triglycerides	mg/dL	Yes	Total
Auto Report	CH	Cholesterol	mg/dL	Yes	Total
Auto Report	L0CH	Cholesterol in LDL, LDL-C	mg/dL	Yes	LDL
Auto Report	H0CH	Cholesterol in HDL, HDL-C	mg/dL	Yes	HDL
Auto Report	ApoA1	Apo-A1	mg/dL	Yes	Total
Auto Report	ApoA2	Apo-A2	mg/dL	Yes	Total
Auto Report	ApoB	Apo-B100	mg/dL	Yes	Total
Auto Report	LHCR	Cholesterol in LDL/Cholesterol in HDL, LDL-C/HDL-C	Ratio	No	Ratio
Auto Report	ABA1R	Apo-B100/Apo-A1	Ratio	No	Ratio
Auto Report	ABPN	Total Apo-B100 Particle Number	nmol/L	Yes	Total
Auto Report	V0PN	VLDL Particle Number	nmol/L	Yes	VLDL
Auto Report	IDPN	IDL Particle Number	nmol/L	Yes	IDL
Auto Report	L0PN	LDL Particle Number	nmol/L	Yes	LDL
Auto Report	L1PN	LDL-1 Particle Number	nmol/L	Yes	LDL
Auto Report	L2PN	LDL-2 Particle Number	nmol/L	Yes	LDL
Auto Report	L3PN	LDL-3 Particle Number	nmol/L	Yes	LDL
Auto Report	L4PN	LDL-4 Particle Number	nmol/L	Yes	LDL
Auto Report	L5PN	LDL-5 Particle Number	nmol/L	Yes	LDL
Auto Report	L6PN	LDL-6 Particle Number	nmol/L	Yes	LDL
Auto Report	V0TG	Triglycerides in VLDL	mg/dL	Yes	VLDL
Auto Report	IDTG	Triglycerides in IDL	mg/dL	Yes	IDL
Auto Report	L0TG	Triglycerides in LDL	mg/dL	Yes	LDL
Auto Report	H0TG	Triglycerides in HDL	mg/dL	Yes	HDL
Auto Report	V0CH	Cholesterol in VLDL	mg/dL	Yes	VLDL
Auto Report	I0CH	Cholesterol in IDL	mg/dL	Yes	IDL
Auto Report	V0FC	Free Cholesterol in VLDL	mg/dL	Yes	VLDL
Auto Report	I0FC	Free Cholesterol in IDL	mg/dL	Yes	IDL
Auto Report	L0FC	Free Cholesterol in LDL	mg/dL	Yes	LDL
Auto Report	H0FC	Free Cholesterol in HDL	mg/dL	Yes	HDL
Auto Report	V0PL	Phospholipids in VLDL	mg/dL	Yes	VLDL
Auto Report	IDPL	Phospholipids in IDL	mg/dL	Yes	IDL
Auto Report	L0PL	Phospholipids in LDL	mg/dL	Yes	LDL
Auto Report	H0PL	Phospholipids in HDL	mg/dL	Yes	HDL
Auto Report	H0A1	Apo-A1 in HDL	mg/dL	Yes	HDL
Auto Report	H0A2	Apo-A2 in HDL	mg/dL	Yes	HDL
Auto Report	V0AB	Apo-B100 in VLDL	mg/dL	Yes	VLDL
Auto Report	IDAB	Apo-B100 in IDL	mg/dL	Yes	IDL
Auto Report	L0AB	Apo-B100 in LDL	mg/dL	Yes	LDL
Auto Report	V1TG	VLDL Subfractions, Triglycerides in VLDL-1	mg/dL	Yes	VLDL
Auto Report	V2TG	VLDL Subfractions, Triglycerides in VLDL-2	mg/dL	Yes	VLDL
Auto Report	V3TG	VLDL Subfractions, Triglycerides in VLDL-3	mg/dL	Yes	VLDL
Auto Report	V4TG	VLDL Subfractions, Triglycerides in VLDL-4	mg/dL	Yes	VLDL
Auto Report	V5TG	VLDL Subfractions, Triglycerides in VLDL-5	mg/dL	Yes	VLDL
Auto Report	V1CH	VLDL Subfractions, Cholesterol in VLDL-1	mg/dL	Yes	VLDL
Auto Report	V2CH	VLDL Subfractions, Cholesterol in VLDL-2	mg/dL	Yes	VLDL
Auto Report	V3CH	VLDL Subfractions, Cholesterol in VLDL-3	mg/dL	Yes	VLDL
Auto Report	V4CH	VLDL Subfractions, Cholesterol in VLDL-4	mg/dL	Yes	VLDL

Auto Report	V5CH	VLDL Subfractions, Cholesterol in VLDL-5	mg/dL	Yes	VLDL
Auto Report	V1FC	VLDL Subfractions, Free Cholesterol in VLDL-1	mg/dL	Yes	VLDL
Auto Report	V2FC	VLDL Subfractions, Free Cholesterol in VLDL-2	mg/dL	Yes	VLDL
Auto Report	V3FC	VLDL Subfractions, Free Cholesterol in VLDL-3	mg/dL	Yes	VLDL
Auto Report	V4FC	VLDL Subfractions, Free Cholesterol in VLDL-4	mg/dL	Yes	VLDL
Auto Report	V5FC	VLDL Subfractions, Free Cholesterol in VLDL-5	mg/dL	Yes	VLDL
Auto Report	V1PL	VLDL Subfractions, Phospholipids in VLDL-1	mg/dL	Yes	VLDL
Auto Report	V2PL	VLDL Subfractions, Phospholipids in VLDL-2	mg/dL	Yes	VLDL
Auto Report	V3PL	VLDL Subfractions, Phospholipids in VLDL-3	mg/dL	Yes	VLDL
Auto Report	V4PL	VLDL Subfractions, Phospholipids in VLDL-4	mg/dL	Yes	VLDL
Auto Report	V5PL	VLDL Subfractions, Phospholipids in VLDL-5	mg/dL	Yes	VLDL
Auto Report	L1TG	LDL Subfractions, Triglycerides in LDL-1	mg/dL	Yes	LDL
Auto Report	L2TG	LDL Subfractions, Triglycerides in LDL-2	mg/dL	Yes	LDL
Auto Report	L3TG	LDL Subfractions, Triglycerides in LDL-3	mg/dL	Yes	LDL
Auto Report	L4TG	LDL Subfractions, Triglycerides in LDL-4	mg/dL	Yes	LDL
Auto Report	L5TG	LDL Subfractions, Triglycerides in LDL-5	mg/dL	Yes	LDL
Auto Report	L6TG	LDL Subfractions, Triglycerides in LDL-6	mg/dL	Yes	LDL
Auto Report	L1CH	LDL Subfractions, Cholesterol in LDL-1	mg/dL	Yes	LDL
Auto Report	L2CH	LDL Subfractions, Cholesterol in LDL-2	mg/dL	Yes	LDL
Auto Report	L3CH	LDL Subfractions, Cholesterol in LDL-3	mg/dL	Yes	LDL
Auto Report	L4CH	LDL Subfractions, Cholesterol in LDL-4	mg/dL	Yes	LDL
Auto Report	L5CH	LDL Subfractions, Cholesterol in LDL-5	mg/dL	Yes	LDL
Auto Report	L6CH	LDL Subfractions, Cholesterol in LDL-6	mg/dL	Yes	LDL
Auto Report	L1FC	LDL Subfractions, Free Cholesterol in LDL-1	mg/dL	Yes	LDL
Auto Report	L2FC	LDL Subfractions, Free Cholesterol in LDL-2	mg/dL	Yes	LDL
Auto Report	L3FC	LDL Subfractions, Free Cholesterol in LDL-3	mg/dL	Yes	LDL
Auto Report	L4FC	LDL Subfractions, Free Cholesterol in LDL-4	mg/dL	Yes	LDL
Auto Report	L5FC	LDL Subfractions, Free Cholesterol in LDL-5	mg/dL	Yes	LDL
Auto Report	L6FC	LDL Subfractions, Free Cholesterol in LDL-6	mg/dL	Yes	LDL
Auto Report	L1PL	LDL Subfractions, Phospholipids in LDL-1	mg/dL	Yes	LDL
Auto Report	L2PL	LDL Subfractions, Phospholipids in LDL-2	mg/dL	Yes	LDL
Auto Report	L3PL	LDL Subfractions, Phospholipids in LDL-3	mg/dL	Yes	LDL
Auto Report	L4PL	LDL Subfractions, Phospholipids in LDL-4	mg/dL	Yes	LDL
Auto Report	L5PL	LDL Subfractions, Phospholipids in LDL-5	mg/dL	Yes	LDL
Auto Report	L6PL	LDL Subfractions, Phospholipids in LDL-6	mg/dL	Yes	LDL
Auto Report	L1AB	LDL Subfractions, Apo B in LDL-1	mg/dL	Yes	LDL
Auto Report	L2AB	LDL Subfractions, Apo B in LDL-2	mg/dL	Yes	LDL
Auto Report	L3AB	LDL Subfractions, Apo B in LDL-3	mg/dL	Yes	LDL
Auto Report	L4AB	LDL Subfractions, Apo B in LDL-4	mg/dL	Yes	LDL
Auto Report	L5AB	LDL Subfractions, Apo B in LDL-5	mg/dL	Yes	LDL
Auto Report	L6AB	LDL Subfractions, Apo B in LDL-6	mg/dL	Yes	LDL
Auto Report	H1TG	HDL Subfractions, Triglycerides in HDL-1	mg/dL	Yes	HDL
Auto Report	H2TG	HDL Subfractions, Triglycerides in HDL-2	mg/dL	Yes	HDL
Auto Report	H3TG	HDL Subfractions, Triglycerides in HDL-3	mg/dL	Yes	HDL
Auto Report	H4TG	HDL Subfractions, Triglycerides in HDL-4	mg/dL	Yes	HDL
Auto Report	H1CH	HDL Subfractions, Cholesterol in HDL-1	mg/dL	Yes	HDL
Auto Report	H2CH	HDL Subfractions, Cholesterol in HDL-2	mg/dL	Yes	HDL
Auto Report	H3CH	HDL Subfractions, Cholesterol in HDL-3	mg/dL	Yes	HDL
Auto Report	H4CH	HDL Subfractions, Cholesterol in HDL-4	mg/dL	Yes	HDL
Auto Report	H1FC	HDL Subfractions, Free Cholesterol in HDL-1	mg/dL	Yes	HDL
Auto Report	H2FC	HDL Subfractions, Free Cholesterol in HDL-2	mg/dL	Yes	HDL

Auto Report	H3FC	HDL Subfractions, Free Cholesterol in HDL-3	mg/dL	Yes	HDL
Auto Report	H4FC	HDL Subfractions, Free Cholesterol in HDL-4	mg/dL	Yes	HDL
Auto Report	H1PL	HDL Subfractions, Phospholipids in HDL-1	mg/dL	Yes	HDL
Auto Report	H2PL	HDL Subfractions, Phospholipids in HDL-2	mg/dL	Yes	HDL
Auto Report	H3PL	HDL Subfractions, Phospholipids in HDL-3	mg/dL	Yes	HDL
Auto Report	H4PL	HDL Subfractions, Phospholipids in HDL-4	mg/dL	Yes	HDL
Auto Report	H1A1	HDL Subfractions, Apo A1 in HDL-1	mg/dL	Yes	HDL
Auto Report	H2A1	HDL Subfractions, Apo A1 in HDL-2	mg/dL	Yes	HDL
Auto Report	H3A1	HDL Subfractions, Apo A1 in HDL-3	mg/dL	Yes	HDL
Auto Report	H4A1	HDL Subfractions, Apo A1 in HDL-4	mg/dL	Yes	HDL
Auto Report	H1A2	HDL Subfractions, Apo A2 in HDL-1	mg/dL	Yes	HDL
Auto Report	H2A2	HDL Subfractions, Apo A2 in HDL-2	mg/dL	Yes	HDL
Auto Report	H3A2	HDL Subfractions, Apo A2 in HDL-3	mg/dL	Yes	HDL
Auto Report	H4A2	HDL Subfractions, Apo A2 in HDL-4	mg/dL	Yes	HDL
Auto Report	EtOH	Ethanol	mmol/L	Yes	Small
Auto Report	TMAO	Trimethylamine-N-oxide	mmol/L	Yes	Small
Auto Report	2 ABA	2-Aminobutyric acid	mmol/L	Yes	Small
Auto Report	Ala	Alanine	mmol/L	Yes	Small
Auto Report	Asp	Asparagine	mmol/L	Yes	Small
Auto Report	Cra	Creatine	mmol/L	Yes	Small
Auto Report	Cre	Creatinine	mmol/L	Yes	Small
Auto Report	Glu	Glutamic acid	mmol/L	Yes	Small
Auto Report	Gln	Glutamine	mmol/L	Yes	Small
Auto Report	Gly	Glycine	mmol/L	Yes	Small
Auto Report	His	Histidine	mmol/L	Yes	Small
Auto Report	Ile	Isoleucine	mmol/L	Yes	Small
Auto Report	Leu	Leucine	mmol/L	Yes	Small
Auto Report	Lys	Lysine	mmol/L	Yes	Small
Auto Report	Met	Methionine	mmol/L	Yes	Small
Auto Report	DMG	N,N-Dimethylglycine	mmol/L	Yes	Small
Auto Report	Omi	Ornithine	mmol/L	Yes	Small
Auto Report	Phe	Phenylalanine	mmol/L	Yes	Small
Auto Report	Pro	Proline	mmol/L	Yes	Small
Auto Report	Sarc	Sarcosine	mmol/L	Yes	Small
Auto Report	Thr	Threonine	mmol/L	Yes	Small
Auto Report	Tyr	Tyrosine	mmol/L	Yes	Small
Auto Report	Val	Valine	mmol/L	Yes	Small
Auto Report	2HBA	2-Hydroxybutyric acid	mmol/L	Yes	Small
Auto Report	AcOH	Acetic acid	mmol/L	Yes	Small
Auto Report	Citrate	Citric acid	mmol/L	Yes	Small
Auto Report	FA	Formic acid	mmol/L	Yes	Small
Auto Report	Lactate	Lactic acid	mmol/L	Yes	Small
Auto Report	Succ	Succinic acid	mmol/L	Yes	Small
Auto Report	Chol	Choline	mmol/L	Yes	Small
Auto Report	2KG	2-Oxoglutaric acid	mmol/L	Yes	Small
Auto Report	3HB	β-Hydroxybutyrate	mmol/L	Yes	Small
Auto Report	AcAc	Acetoacetate	mmol/L	Yes	Small
Auto Report	Acto	Acetone	mmol/L	Yes	Small
Auto Report	Pyr	Pyruvic acid	mmol/L	Yes	Small
Auto Report	GAL	D-Galactose	mmol/L	Yes	Small
Auto Report	GLC	Glucose	mmol/L	Yes	Small
Auto Report	Glycol	Glycerol	mmol/L	Yes	Small
Auto Report	DMSF	Dimethylsulfone	mmol/L	Yes	Small

Auto Report	CaEDTA	Ca-EDTA	mmol/L	Yes	Small
Auto Report	K-EDTA	K-EDTA	mmol/L	Yes	Small
Calculate	UFAp	Unsaturated fatty acid/Total fatty acid (%)	Percentage	No	Small
Calculate	SFAp	Saturated fatty acid/Total fatty acid (%)	Percentage	No	Small
Calculate	PUFAp	Polyunsaturated fatty acid/Total fatty acid (%)	Percentage	No	Small
Calculate	MUFAp	Monounsaturated fatty acid/Total fatty acid (%)	Percentage	No	Small
Calculate	MUPUR	MUFA to PUFA ratio	Ratio	No	Small
Calculate	UPUR	UFA to PUFA ratio	Ratio	No	Small
Calculate	TGall	Total TG in IDL, VLDL, LDL and HDL	mg/dL	No	Total
Calculate	CHall	Total CH in IDL, VLDL, LDL and HDL	mg/dL	No	Total
Calculate	FC	Total FC in IDL, VLDL, LDL and HDL	mg/dL	No	Total
Calculate	PL	Total PL in IDL, VLDL, LDL and HDL	mg/dL	No	Total
Calculate	CE	Total CE in IDL, VLDL, LDL and HDL	mg/dL	No	Total
Calculate	V0CE	Cholesterol esters in VLDL	mg/dL	No	VLDL
Calculate	IDCE	Cholesterol esters in IDL	mg/dL	No	IDL
Calculate	L0CE	Cholesterol esters in LDL	mg/dL	No	LDL
Calculate	H0CE	Cholesterol esters in HDL	mg/dL	No	HDL
Calculate	V0LP	Total lipids in VLDL	mg/dL	No	VLDL
Calculate	IDLP	Total lipids in IDL	mg/dL	No	IDL
Calculate	L0LP	Total lipids in LDL	mg/dL	No	LDL
Calculate	H0LP	Total lipids in HDL	mg/dL	No	HDL
Calculate	V1CE	VLDL Subfractions, Cholesterol esters in VLDL-1	mg/dL	No	VLDL
Calculate	V2CE	VLDL Subfractions, Cholesterol esters in VLDL-2	mg/dL	No	VLDL
Calculate	V3CE	VLDL Subfractions, Cholesterol esters in VLDL-3	mg/dL	No	VLDL
Calculate	V4CE	VLDL Subfractions, Cholesterol esters in VLDL-4	mg/dL	No	VLDL
Calculate	V5CE	VLDL Subfractions, Cholesterol esters in VLDL-5	mg/dL	No	VLDL
Calculate	L1CE	LDL Subfractions, Cholesterol esters in LDL-1	mg/dL	No	LDL
Calculate	L2CE	LDL Subfractions, Cholesterol esters in LDL-2	mg/dL	No	LDL
Calculate	L3CE	LDL Subfractions, Cholesterol esters in LDL-3	mg/dL	No	LDL
Calculate	L4CE	LDL Subfractions, Cholesterol esters in LDL-4	mg/dL	No	LDL
Calculate	L5CE	LDL Subfractions, Cholesterol esters in LDL-5	mg/dL	No	LDL
Calculate	L6CE	LDL Subfractions, Cholesterol esters in LDL-6	mg/dL	No	LDL
Calculate	H1CE	HDL Subfractions, Cholesterol esters in HDL-1	mg/dL	No	HDL
Calculate	H2CE	HDL Subfractions, Cholesterol esters in HDL-2	mg/dL	No	HDL
Calculate	H3CE	HDL Subfractions, Cholesterol esters in HDL-3	mg/dL	No	HDL
Calculate	H4CE	HDL Subfractions, Cholesterol esters in HDL-4	mg/dL	No	HDL
Calculate	V1LP	VLDL Subfractions, Total lipids in VLDL-1	mg/dL	No	VLDL
Calculate	V2LP	VLDL Subfractions, Total lipids in VLDL-2	mg/dL	No	VLDL
Calculate	V3LP	VLDL Subfractions, Total lipids in VLDL-3	mg/dL	No	VLDL
Calculate	V4LP	VLDL Subfractions, Total lipids in VLDL-4	mg/dL	No	VLDL
Calculate	V5LP	VLDL Subfractions, Total lipids in VLDL-5	mg/dL	No	VLDL
Calculate	L1LP	LDL Subfractions, Total lipids in LDL-1	mg/dL	No	LDL
Calculate	L2LP	LDL Subfractions, Total lipids in LDL-2	mg/dL	No	LDL
Calculate	L3LP	LDL Subfractions, Total lipids in LDL-3	mg/dL	No	LDL
Calculate	L4LP	LDL Subfractions, Total lipids in LDL-4	mg/dL	No	LDL
Calculate	L5LP	LDL Subfractions, Total lipids in LDL-5	mg/dL	No	LDL
Calculate	L6LP	LDL Subfractions, Total lipids in LDL-6	mg/dL	No	LDL
Calculate	H1LP	HDL Subfractions, Total lipids in HDL-1	mg/dL	No	HDL
Calculate	H2LP	HDL Subfractions, Total lipids in HDL-2	mg/dL	No	HDL
Calculate	H3LP	HDL Subfractions, Total lipids in HDL-3	mg/dL	No	HDL
Calculate	H4LP	HDL Subfractions, Total lipids in HDL-4	mg/dL	No	HDL
Calculate	V1TGp	Triglycerides to Total lipids ratio in VLDL-1	Percentage	No	VLDL
Calculate	V1CHp	Cholesterol to Total lipids ratio in VLDL-1	Percentage	No	VLDL

Calculate	V1FCp	Free cholesterol to Total lipids ratio in VLDL-1	Percentage	No	VLDL
Calculate	V1PLp	Phospholipids to Total lipids ratio in VLDL-1	Percentage	No	VLDL
Calculate	V2TGp	Triglycerides to Total lipids ratio in VLDL-2	Percentage	No	VLDL
Calculate	V2CHp	Cholesterol to Total lipids ratio in VLDL-2	Percentage	No	VLDL
Calculate	V2FCp	Free cholesterol to Total lipids ratio in VLDL-2	Percentage	No	VLDL
Calculate	V2PLp	Phospholipids to Total lipids ratio in VLDL-2	Percentage	No	VLDL
Calculate	V3TGp	Triglycerides to Total lipids ratio in VLDL-3	Percentage	No	VLDL
Calculate	V3CHp	Cholesterol to Total lipids ratio in VLDL-3	Percentage	No	VLDL
Calculate	V3FCp	Free cholesterol to Total lipids ratio in VLDL-3	Percentage	No	VLDL
Calculate	V3PLp	Phospholipids to Total lipids ratio in VLDL-3	Percentage	No	VLDL
Calculate	V4TGp	Triglycerides to Total lipids ratio in VLDL-4	Percentage	No	VLDL
Calculate	V4CHp	Cholesterol to Total lipids ratio in VLDL-4	Percentage	No	VLDL
Calculate	V4FCp	Free cholesterol to Total lipids ratio in VLDL-4	Percentage	No	VLDL
Calculate	V4PLp	Phospholipids to Total lipids ratio in VLDL-4	Percentage	No	VLDL
Calculate	V5TGp	Triglycerides to Total lipids ratio in VLDL-5	Percentage	No	VLDL
Calculate	V5CHp	Cholesterol to Total lipids ratio in VLDL-5	Percentage	No	VLDL
Calculate	V5FCp	Free cholesterol to Total lipids ratio in VLDL-5	Percentage	No	VLDL
Calculate	V5PLp	Phospholipids to Total lipids ratio in VLDL-5	Percentage	No	VLDL
Calculate	L1TGp	Triglycerides to Total lipids ratio in LDL-1	Percentage	No	LDL
Calculate	L1CHp	Cholesterol to Total lipids ratio in LDL-1	Percentage	No	LDL
Calculate	L1FCp	Free cholesterol to Total lipids ratio in LDL-1	Percentage	No	LDL
Calculate	L1PLp	Phospholipids to Total lipids ratio in LDL-1	Percentage	No	LDL
Calculate	L2TGp	Triglycerides to Total lipids ratio in LDL-2	Percentage	No	LDL
Calculate	L2CHp	Cholesterol to Total lipids ratio in LDL-2	Percentage	No	LDL
Calculate	L2FCp	Free cholesterol to Total lipids ratio in LDL-2	Percentage	No	LDL
Calculate	L2PLp	Phospholipids to Total lipids ratio in LDL-2	Percentage	No	LDL
Calculate	L3TGp	Triglycerides to Total lipids ratio in LDL-3	Percentage	No	LDL
Calculate	L3CHp	Cholesterol to Total lipids ratio in LDL-3	Percentage	No	LDL
Calculate	L3FCp	Free cholesterol to Total lipids ratio in LDL-3	Percentage	No	LDL
Calculate	L3PLp	Phospholipids to Total lipids ratio in LDL-3	Percentage	No	LDL
Calculate	L4TGp	Triglycerides to Total lipids ratio in LDL-4	Percentage	No	LDL
Calculate	L4CHp	Cholesterol to Total lipids ratio in LDL-4	Percentage	No	LDL
Calculate	L4FCp	Free cholesterol to Total lipids ratio in LDL-4	Percentage	No	LDL
Calculate	L4PLp	Phospholipids to Total lipids ratio in LDL-4	Percentage	No	LDL
Calculate	L5TGp	Triglycerides to Total lipids ratio in LDL-5	Percentage	No	LDL
Calculate	L5CHp	Cholesterol to Total lipids ratio in LDL-5	Percentage	No	LDL
Calculate	L5FCp	Free cholesterol to Total lipids ratio in LDL-5	Percentage	No	LDL
Calculate	L5PLp	Phospholipids to Total lipids ratio in LDL-5	Percentage	No	LDL
Calculate	L6TGp	Triglycerides to Total lipids ratio in LDL-6	Percentage	No	LDL
Calculate	L6CHp	Cholesterol to Total lipids ratio in LDL-6	Percentage	No	LDL
Calculate	L6FCp	Free cholesterol to Total lipids ratio in LDL-6	Percentage	No	LDL
Calculate	L6PLp	Phospholipids to Total lipids ratio in LDL-6	Percentage	No	LDL
Calculate	H1TGp	Triglycerides to Total lipids ratio in HDL-1	Percentage	No	HDL
Calculate	H1CHp	Cholesterol to Total lipids ratio in HDL-1	Percentage	No	HDL
Calculate	H1FCp	Free cholesterol to Total lipids ratio in HDL-1	Percentage	No	HDL
Calculate	H1PLp	Phospholipids to Total lipids ratio in HDL-1	Percentage	No	HDL
Calculate	H2TGp	Triglycerides to Total lipids ratio in HDL-2	Percentage	No	HDL
Calculate	H2CHp	Cholesterol to Total lipids ratio in HDL-2	Percentage	No	HDL
Calculate	H2FCp	Free cholesterol to Total lipids ratio in HDL-2	Percentage	No	HDL
Calculate	H2PLp	Phospholipids to Total lipids ratio in HDL-2	Percentage	No	HDL
Calculate	H3TGp	Triglycerides to Total lipids ratio in HDL-3	Percentage	No	HDL
Calculate	H3CHp	Cholesterol to Total lipids ratio in HDL-3	Percentage	No	HDL
Calculate	H3FCp	Free cholesterol to Total lipids ratio in HDL-3	Percentage	No	HDL

Calculate	H3PLp	Phospholipids to Total lipids ratio in HDL-3	Percentage	No	HDL
Calculate	H4TGp	Triglycerides to Total lipids ratio in HDL-4	Percentage	No	HDL
Calculate	H4CHp	Cholesterol to Total lipids ratio in HDL-4	Percentage	No	HDL
Calculate	H4FCp	Free cholesterol to Total lipids ratio in HDL-4	Percentage	No	HDL
Calculate	H4PLp	Phospholipids to Total lipids ratio in HDL-4	Percentage	No	HDL
Calculate	V1CEp	Cholesterol esters to Total lipids ratio in VLDL-1	Percentage	No	VLDL
Calculate	V2CEp	Cholesterol esters to Total lipids ratio in VLDL-2	Percentage	No	VLDL
Calculate	V3CEp	Cholesterol esters to Total lipids ratio in VLDL-3	Percentage	No	VLDL
Calculate	V4CEp	Cholesterol esters to Total lipids ratio in VLDL-4	Percentage	No	VLDL
Calculate	V5CEp	Cholesterol esters to Total lipids ratio in VLDL-5	Percentage	No	VLDL
Calculate	L1CEp	Cholesterol esters to Total lipids ratio in LDL-1	Percentage	No	LDL
Calculate	L2CEp	Cholesterol esters to Total lipids ratio in LDL-2	Percentage	No	LDL
Calculate	L3CEp	Cholesterol esters to Total lipids ratio in LDL-3	Percentage	No	LDL
Calculate	L4CEp	Cholesterol esters to Total lipids ratio in LDL-4	Percentage	No	LDL
Calculate	L5CEp	Cholesterol esters to Total lipids ratio in LDL-5	Percentage	No	LDL
Calculate	L6CEp	Cholesterol esters to Total lipids ratio in LDL-6	Percentage	No	LDL
Calculate	H1CEp	Cholesterol esters to Total lipids ratio in HDL-1	Percentage	No	HDL
Calculate	H2CEp	Cholesterol esters to Total lipids ratio in HDL-2	Percentage	No	HDL
Calculate	H3CEp	Cholesterol esters to Total lipids ratio in HDL-3	Percentage	No	HDL
Calculate	H4CEp	Cholesterol esters to Total lipids ratio in HDL-4	Percentage	No	HDL
Calculate	V0CEp	Cholesterol esters to Total lipids ratio in VLDL	Percentage	No	VLDL
Calculate	IDCEp	Cholesterol esters to Total lipids ratio in IDL	Percentage	No	IDL
Calculate	L0CEp	Cholesterol esters to Total lipids ratio in LDL	Percentage	No	LDL
Calculate	HDCEp	Cholesterol esters to Total lipids ratio in HDL	Percentage	No	HDL
Calculate	V0TGp	Triglycerides to Total lipids ratio in VLDL	Percentage	No	VLDL
Calculate	IDTGp	Triglycerides to Total lipids ratio in IDL	Percentage	No	IDL
Calculate	L0TGp	Triglycerides to Total lipids ratio in LDL	Percentage	No	LDL
Calculate	H0TGp	Triglycerides to Total lipids ratio in HDL	Percentage	No	HDL
Calculate	V0CHp	Cholesterol to Total lipids ratio in VLDL	Percentage	No	VLDL
Calculate	IDCHp	Cholesterol to Total lipids ratio in IDL	Percentage	No	IDL
Calculate	L0CHp	Cholesterol to Total lipids ratio in LDL	Percentage	No	LDL
Calculate	H0CHp	Cholesterol to Total lipids ratio in HDL	Percentage	No	HDL
Calculate	V0FCp	Free cholesterol to Total lipids ratio in VLDL	Percentage	No	VLDL
Calculate	IDFCp	Free cholesterol to Total lipids ratio in IDL	Percentage	No	IDL
Calculate	L0FCp	Free cholesterol to Total lipids ratio in LDL	Percentage	No	LDL
Calculate	H0FCp	Free cholesterol to Total lipids ratio in HDL	Percentage	No	HDL
Calculate	V0PLp	Phospholipids to Total lipids ratio in VLDL	Percentage	No	VLDL
Calculate	IDPLp	Phospholipids to Total lipids ratio in IDL	Percentage	No	IDL
Calculate	L0PLp	Phospholipids to Total lipids ratio in LDL	Percentage	No	LDL
Calculate	H0PLp	Phospholipids to Total lipids ratio in HDL	Percentage	No	HDL
Calculate	TGPLR	Triglycerides to Phospholipid ratio	Ratio	No	Ratio
Calculate	nH0CH	non-HDL Cholesterol, non-HDL-C	nmol/L	No	Total
Calculate	nH0CHp	non-HDL Cholesterol/Cholesterol, non-HDL-C/CH	Ratio	No	Total
Calculate	CH/HC	Cholesterol to HDL-C ratio	Ratio	No	Ratio
Calculate	AB/HC	Apo-B100 to HDL-C ratio	Ratio	No	Ratio
Calculate	TG/HC	Triglycerides to HDL-C ratio	Ratio	No	Ratio
Calculate	VCHHCR	Cholesterol in VLDL to Cholesterol in HDL ratio	Ratio	No	Ratio
Calculate	ICHHCR	Cholesterol in IDL to Cholesterol in HDL ratio	Ratio	No	Ratio
Calculate	VLCHHCR	Cholesterol in (VLDL + LDL) to Cholesterol in HDL ratio	Ratio	No	Ratio
Calculate	nHCHCR	Cholesterol in (VLDL + IDL + LDL) to Cholesterol in HDL ratio	Ratio	No	Ratio
Calculate	L1PNp	LDL-1/LDL Particle Number	Percentage	No	LDL

Calculate	L2PNp	LDL-2/LDL Particle Number	Percentage	No	LDL
Calculate	L3PNp	LDL-3/LDL Particle Number	Percentage	No	LDL
Calculate	L4PNp	LDL-4/LDL Particle Number	Percentage	No	LDL
Calculate	L5PNp	LDL-5/LDL Particle Number	Percentage	No	LDL
Calculate	L6PNp	LDL-6/LDL Particle Number	Percentage	No	LDL
Calculate	CHTGR	Cholesterol to Triglycerides ratio	Ratio	No	Ratio
Calculate	V0CHTGR	Cholesterol to Triglycerides ratio in VLDL	Ratio	No	Ratio
Calculate	V1CHTGR	Cholesterol to Triglycerides ratio in VLDL-1	Ratio	No	Ratio
Calculate	V2CHTGR	Cholesterol to Triglycerides ratio in VLDL-2	Ratio	No	Ratio
Calculate	V3CHTGR	Cholesterol to Triglycerides ratio in VLDL-3	Ratio	No	Ratio
Calculate	V4CHTGR	Cholesterol to Triglycerides ratio in VLDL-4	Ratio	No	Ratio
Calculate	V5CHTGR	Cholesterol to Triglycerides ratio in VLDL-5	Ratio	No	Ratio
Calculate	IDCHTGR	Cholesterol to Triglycerides ratio in IDL	Ratio	No	Ratio
Calculate	L0CHTGR	Cholesterol to Triglycerides ratio in LDL	Ratio	No	Ratio
Calculate	L1CHTGR	Cholesterol to Triglycerides ratio in LDL-1	Ratio	No	Ratio
Calculate	L2CHTGR	Cholesterol to Triglycerides ratio in LDL-2	Ratio	No	Ratio
Calculate	L3CHTGR	Cholesterol to Triglycerides ratio in LDL-3	Ratio	No	Ratio
Calculate	L4CHTGR	Cholesterol to Triglycerides ratio in LDL-4	Ratio	No	Ratio
Calculate	L5CHTGR	Cholesterol to Triglycerides ratio in LDL-5	Ratio	No	Ratio
Calculate	L6CHTGR	Cholesterol to Triglycerides ratio in LDL-6	Ratio	No	Ratio
Calculate	H0CHTGR	Cholesterol to Triglycerides ratio in HDL	Ratio	No	Ratio
Calculate	H1CHTGR	Cholesterol to Triglycerides ratio in HDL-1	Ratio	No	Ratio
Calculate	H2CHTGR	Cholesterol to Triglycerides ratio in HDL-2	Ratio	No	Ratio
Calculate	H3CHTGR	Cholesterol to Triglycerides ratio in HDL-3	Ratio	No	Ratio
Calculate	H4CHTGR	Cholesterol to Triglycerides ratio in HDL-4	Ratio	No	Ratio
Calculate	VIDPN	Particle Number in (VLDL+IDL)	nmol/L	No	Total
Calculate	VIDTG	Triglycerides in (VLDL+IDL)	nmol/L	No	Total
Calculate	VIDCH	Cholesterol in (VLDL+IDL)	nmol/L	No	Total
Calculate	VIDFC	Free Cholesterol in (VLDL+IDL)	nmol/L	No	Total
Calculate	VIDPL	Phospholipids in (VLDL+IDL)	nmol/L	No	Total
Calculate	VIDAB	Apo-B in (VLDL+IDL)	nmol/L	No	Total
Calculate	VILPN	Particle Number in (VLDL+IDL+LDL)	nmol/L	No	Total
Calculate	VILTG	Triglycerides in (VLDL+IDL+LDL)	nmol/L	No	Total
Calculate	VILCH	Cholesterol in (VLDL+IDL+LDL)	nmol/L	No	Total
Calculate	VILFC	Free Cholesterol in (VLDL+IDL+LDL)	nmol/L	No	Total
Calculate	VILPL	Phospholipids in (VLDL+IDL+LDL)	nmol/L	No	Total
Calculate	VILAB	Apo-B in (VLDL+IDL+LDL)	nmol/L	No	Total
Calculate	LactPyR	Lactate to Pyruvate ratio	Ratio	No	Small
Calculate	FisherR	Branched-chain amino acids (Leu+Val+Ile) to Aromatic amino acids (Phe+Tyr+Trp) ratio	Ratio	No	Small
Calculate	3HB/AcAc	β-Hydroxybutyrate to acetoacetate ratio	Ratio	No	Small
Calculate	NAG1	N-acetylglucosamine/N-acetylgalactosamine-glycoproteins (acute phase marker for inflammation)	Peak Area	Yes	Small
Calculate	NAG2	N-acetylneuraminoyl-glycoproteins (acute phase marker for inflammation)	Peak Area	Yes	Small
Calculate	NAG12R	NAG1 to NAG2 ratio	Ratio	No	Small
Calculate	DHT	Dihydrothymine	mmol/L	Yes	Small
Calculate	Mg	Mg-EDTA (plasma only)	mmol/L	Yes	Small
Calculate	GluA	Glutamic acid (peak area)	Peak Area	Yes	Small
Calculate	GlnA	Glutamine (peak area)	Peak Area	Yes	Small
